# Effects of COVID-19 pandemic lockdowns on college students’ physical fitness: A comparative study

**DOI:** 10.1371/journal.pone.0335309

**Published:** 2025-11-04

**Authors:** Jianzhong Sun, Minghao Wu, Jun Li, Cunjian Bi

**Affiliations:** 1 School of Physical Education, Chizhou University, Chizhou, Anhui, China; 2 Sports for Health Promotion Center, Chizhou University, Chizhou, Anhui, China; Galgotias University, INDIA

## Abstract

**Background:**

The impact of the COVID-19 outbreak has been significant and far-reaching. This paper retrospectively examines its effects on changes in the physical fitness of university students.

**Method:**

From 2018 to 2020, a comprehensive undergraduate institution in southern Anhui Province was selected to assess the impact of the COVID-19 pandemic on physical health indicators among college students.

**Results:**

The overall physical fitness of students at Chizhou University was clustered near the passing threshold of 80.8%, with 13.0% of students failing to meet the standard. The COVID-19 pandemic contributed to an increased prevalence of overweight (11.3%) and obesity (4.4%) among the student population. In endurance running, both male and female students displayed a similar trend—initial improvement followed by a decline—resulting in reduced performance times by 16.7 s for males and 13.2 s for females in 2020. Notably, vital capacity and upper limb strength showed consistent upward trends from 2018 to 2020 for both genders. Specifically, male students’ vital capacity increased by 2.0% and 3.4%, and upper limb strength by 4.8% and 8.3%, in consecutive years. Similarly, female students’ vital capacity rose by 1.3% and 4.4%, while their upper limb strength improved by 3.9% and 3.5%, respectively.

**Conclusion:**

The short-term impact of the COVID-19 epidemic significant effected college students’ endurance running performance. Future efforts should focus on the important long-term lagging effects of the pandemic. Meanwhile, there is an urgent need to better understand and develop alternative exercise modalities during major public health events to ensure the maintenance of physical fitness and overall health.

## Introduction

The COVID-19 pandemic has profoundly impacted daily life and well-being worldwide [1–3]. Government-imposed lockdowns and related restrictions significantly reduced opportunities for physical activity, resulting in a 28.0% increase in average daily sitting time [4]. This global crisis has further worsened the already declining physical health of college students worldwide [5]. According to López-Valenciano et al., college students exhibited reductions in walking, moderate, strenuous, and total physical activity [6]. Such decline in activity levels has also linked to increased psychological stress among students [7]. The consequences of this decline are both severe and multifaceted. Poor physical fitness often exacerbated by substantial shifts in lifestyle and learning modalities during the pandemic [8], which may intensify weight-related shame among students [9]. This, in turn, increases psychological [10], leads to reduced physical activity, prolongs sedentary behavior [11,12], and raises the consumption of unhealthy foods [13,14], forming a self-perpetuating cycle that further worsens physical health. Studies have shown a positive correlation between low physical fitness and poor academic performance or negative emotions [15,16]. Sports participation has been shown to improve life satisfaction [17,18], while increased physical activity can strengthen psychological resilience and enhance social adaptability after graduation [19].

In January 2020, the World Health Organization issued a global alert, prompting the Chinese government to implement measures such as city lockdowns and the closure of public spaces [1–4,11]. Given their dense populations, colleges and universities were crucial in epidemic prevention and control [2,3]. At the beginning of the spring 2020 semester, most institutions rapidly switched to online classes, covering both theoretical courses across various disciplines and physical education [7,17]. Theoretical courses were delivered remotely, with substitute instructions taking over teaching duties. For physical education, instructors emphasized organizing indoor activities and encouraged students to independently achieve recommended exercise volume and duration [18,19]. Concurrently, students’ lifestyles underwent dramatic changes. Confined to campus and unable to move freely, students increasingly relied on food delivery services, leading to a sharp rise in takeaway consumption. Additionally, screen time—for both online courses and entertainment—increased significantly. These shifts imposed unprecedented mental and psychological pressure on students [20,21].

Therefore, it is essential to monitor and address the current physical fitness status of college students [22]. This study has two main objectives. First, it seeks to gather baseline data on the physical fitness, health status, and dynamic characteristics of college students during COVID-19 epidemic, thereby enriching the database on the impact of the COVID-19 pandemic on the physical health of college students in China. Second, it investigates changes in the physical fitness of students at Chizhou University before and after the onset of the COVID-19 outbreak, providing athorough analysis of how shifts in lifestyle and learing models have influenced students health.

## Methods

### Study participants

All students from a comprehensive undergraduate institution in southern Anhui Province were selected as the study sample, encompassing four grades (freshmen to seniors) across 11 academic majors in both arts and sciences. A retrospective analysis was conducted on physical fitness test data from 2018 to 2020. Although graduation and new enrollment occurred each year in July and September, the entire student body was treated as a single cohort throughout the study period to more accurately reflect the impact of the pandemic. Furthermore, the physical fitness tests were scheduled for late October each year, avoiding the periods of graduation and enrollment, with the aim of collecting more reliable and precise data.

To assess the impact of the COVID-19 pandemic on the physical fitness of university students, participants were included if they were fully enrolled and physically healthy. Exclusion criteria consisted of any physical or mental health conditions that could prevent completion of all physical fitness tests. After removing outliers and accounting for temporary absences during testing (totaling 5.3% of the initial sample), a final dataset of 42,391 participant records was included in the analysis. Detailed information is presented in Table 1.

**Table 1:** Statistics of sample size in this study.

Grade	Year		
**2018**	**2019**	**2020**
freshman	3,471	3,574	4,019
sophomore	3,445	3,495	3,935
junior	3,496	3,408	3,473
senior	3,398	3,304	3,373
Total	13,810	13,781	14,800

The participant recruitment period for this study spanned from September 1, 2018 to November, 1, 2020. As a result, the physical fitness data from 2018 and 2019 were collected prior to the COVID-19 pandemic and were thus unaffected by the epidemic. In contrast, the data from 2020 were obtained during the pandemic and therefore reflect of COVID-19 pandemic, including significant changes in lifestyle and learing patterns.

### Ethical approval and informed consent

All study protocols were approved by the Human Experimental Ethics Committee of Chizhou University (Approval Nos: CZ2018YJRC01 and CZ2019YJRC01). Written informed consent was obtained from all participants, with additional parental consent provided for those aged 17 years or younger, prior to each physical fitness assessment.

### Data collection of physical fitness indicators

The physical fitness assessment comprised the following indicators: body composition (height and weight), flexibility (sit-and-reach test), upper limb strength (pull-ups for males and sit-ups for females), lower limb strength (standing long jump and 50-m dash), and cardiopulmonary endurance (vital capacity, 1000 m run for males, and 800 m run for females). The testing protocols for these indicators adhered to the methods described in our previous publication [23]. Additional details regarding tests and equipment can be found in another of our reference [24].

All physical fitness indicators were assessed in strict accordance with the National Student Physical Health Standard of China (2014 edition) [25]. The tests were conducted annually in late October by a professional team consisting of faculty and students from the institute of physical education. Following standard research protocols, one week prior to testing, all participant students received detailed instructions regarding the test procedures, while staff members underwent formal training on assessment protocols. Equipments were calibrated daily at 7:00 AM and at regular intervals throughout the testing day—specifically at 8:00 AM, 10:00 AM, 1:00 PM, 3:00 PM, and 5:00 PM (at lease five times per day). Formal testsing sessions were held daily from 8:00 AM to 11:30 AM and from 2:00 PM to 5:30 PM.

### Data analysis

All physical fitness indicators were scored according to the National Student Physical Health Standard of China (2014 edition) and analyzed using SPSS software version 25.0 (SPSS Inc., Chicago, IL, USA). The median (interquartile range) was used to quantify the physical fitness test results from 2018 to 2020, including height (cm), weight (kg), BMI (kg/m²), vital capacity (ml), standing long jump (cm), sit-and-reach (cm), 50-m dash (s), pull-ups (count), 1000 m run (s), sit-ups (count), 800 m run (s), and total score. Data normality was assessed using the Kolmogorov-Smirnov test. Differences in each physical fitness indicator across the three years were compared using the Kruskal-Wallis H test, with Bonferroni adjustment applied for-post hoc analyses. A *p*- value< 0.05 was considered statistically significant.

The calssification criteria were defined as follows. For BMI, underweight (< 18.5), normal weight (18.5 ≤ BMI < 23.9), overweight (24.0 ≤ BMI < 27.9), and obesity (≥ 28.0). For the 1000 m/800 m (in seconds), excellent (≤207 for male, ≤ 210 for female), good (≤222 and >207 for male, > 210 and ≤224 for female), pass (≤272 and >222 for male, > 224 and ≤274 for female), and fail (>272 for male, > 274 for female). For the total score, excellent(≥90), good (≥80 and ≤89.9), pass (≥60 and ≤79.9), and fail (≤59.9).

## Results

The results of the physical fitness indicators and comparative analyses of college students from 2018 to 2020 are summarized in Table 2 (see supplementary material S1 Table for detailed categorical statistics of other physical test items). This table provides annual median and interquartile range for each test item, stratified by gender, to facilitate the identification of significant inter annusl differences. Notably, significant differences were observed between male and female students from 2018 to 2020 in height, vital capacity, upper limb strength (pull-ups for males and sit-ups for females), endurance running (1000 m for males and 800 m for females), and overall physical fitness score. Endurance running, in particular, showed a consistent trend across both genders: an initial improvement followed by a subsequent decline.

**Table 2:** Median (interquartile range) and difference comparisonof ten physical fitness indexes by gender.

Gender	Items	2018(A)	2019(B)	2020(C)	A-B	A-C	B-C
Male	Height (cm)	173.9(7.7)	174.0(8.0)	175.0(7.0)	<0.001*	<0.001*	<0.001*
	Weight (kg)	65.0(13.4)	65.0(12.5)	65.0(15.0)	0.8	<0.001*	<0.001*
	BMI (kg/m^2^)	21.5(4.3)	21.4(4.4)	21.5(4.4)	0.2		
	Vital capacity (ml)	3,583.0(916.3)	3,646.5(820.8)	3,729.5(818.5)	<0.001*	<0.001*	<0.001*
	Standing long jump (cm)	229.0(30.0)	225.0(30.0)	225.0(22.0)	0.2	0.2	0.001*
	Sit-and-reach (cm)	13.0(8.8)	13.2(8.5)	12.5(7.5)	<0.001*	1.0	<0.001*
	50-m dash (s)	7.5(1.0)	7.5(0.9)	7.5(0.9)	<0.001*	0.4	<0.001*
	Pull-ups (count)	6.0(6.0)	7.0(6.0)	7.0(6.0)	<0.001*	<0.001*	<0.001*
	1000 m (s)	257.0(37.0)	252.0(31.0)	268.0(49.0)	<0.001*	<0.001*	<0.001*
	Total score	66.2(11.6)	68.3(11.1)	67.5(11.0)	<0.001*	<0.001*	<0.001*
Female	Height (cm)	161.6(7.0)	162.0(6.0)	163.0(6.0)	<0.001*	<0.001*	<0.001*
	Weight (kg)	52.3(9.1)	53.0(9.3)	52.0(9.0)	0.06	0.07	<0.001*
	BMI (kg/m^2^)	20.1(3.1)	20.0(3.1)	19.6(3.1)	1.0	<0.001*	<0.001*
	Vital capacity (ml)	2,399.5(582.0)	2,420.0(548.0)	2,518.0(582.8)	<0.001*	<0.001*	<0.001*
	Standing long jump (cm)	170.0(20.0)	170.0(20.0)	170.0(20.0)	0.6		
	Sit-and-reach (cm)	16.4(7.0)	17.0(7.0)	17.0(7.0)	0.001*	1.0	0.006*
	50-m dash (s)	9.4(1.2)	9.2(1.2)	9.3(1.0)	<0.001*	<0.001*	<0.001*
	Sit-ups (count)	30.0(9.0)	31.0(7.0)	32.0(7.0)	<0.001*	<0.001*	<0.001*
	800 m (s)	249.0(27.0)	244.0(27.0)	257.0(33.0)	<0.001*	<0.001*	<0.001*
	Total score	70.6(10.3)	72.8(8.4)	72.0(7.4)	<0.001*	<0.001*	<0.001*

Note: A-B, A-C, B-C mean the *p*-values of Kruskal-Wallis H test (two-tailed) between 2018–2019, 2018–2020, and 2019–2020; *p < 0.05.

A total of 42,391 students (17,830 males and 24,561 females) were included in this study. Table 3 presents the age distribution, BMI categories, endurance running grades, and overall physical test scores of the participants from 2018 to 2020. As shown in Table 3, approximately 14,000 students were assessed each year, with a slightly higher proportion of female students in the mid-study period. The age of participants predominantly ranged from 18 to 23 years. Regarding BMI, the majority of students (77.0%) fell within the normal weight range, with a significantly higher proportion among female students (83.4%) compared to males (68.1%). Overweight individuals constituted the second-largest group with an average of 11.3% over the three years, and were more prevalent among males (17.3%) than females (7.0%). The underweight group comprised 7.2% of the total population, with similar rates between genders (males: 7.2%; females: 7.1%). Obese individuals represented an average of 4.4% across there years, with a notably higher rate among males (7.3%) than females (2.3%).

**Table 3:** Data of age distribution and main physique of the subjects in this study, N (%).

Variables		Total			Male			Female		
		2018	2019	2020	2018	2019	2020	2018	2019	2020
Age(years)	≤17	227(1.6)	218(1.5)	231(1.5)	82(1.4)	83(1.4)	90(1.4)	145(1.7)	135(1.6)	141(0.6)
	18	1,685(12.2)	1,750(12.6)	1,784(12.0)	649(11.3)	650(11.3)	759(11.9)	1,036(12.8)	1,100(13.6)	1,025(12.1)
	19	2,759(19.9)	2,992(21.7)	2,965(20.0)	1,087(18.9)	1,247(21.7)	1,192(18.7)	1,672(20.6)	1,745(21.7)	1,773(21.0)
	20	3,089(22.3)	3,168(22.9)	3,430(23.1)	1,237(21.5)	1,284(22.3)	1,483(23.3)	1,852(22.9)	1,884(23.4)	1,947(23.0)
	21	3,060(22.1)	2,931(21.2)	3,247(21.9)	1,256(21.9)	1,206(21.0)	1,350(21.2)	1,804(22.3)	1,725(21.4)	1,897(22.4)
	22	1,901(13.7)	1,796(13.0)	2,026(13.6)	865(15.0)	799(13.9)	931(14.6)	1,036(12.8)	997(12.3)	1,095(12.9)
	23	790(5.7)	665(4.8)	827(5.5)	393(6.8)	319(5.5)	400(6.2)	397(4.9)	346(4.3)	427(5.0)
	≥24	299(2.1)	261(1.8)	290(1.9)	161(2.8)	152(2.6)	155(2.4)	138(1.7)	109(1.3)	135(1.5)
Total participant		13,810	13,781	14,800	5,730	5,740	6,360	8,080	8,041	8,440
BMI Status	Underweight	937(6.7)	925(6.7)	1,201(8.1)	423(7.3)	421(7.3)	448(7.0)	514(6.3)	504(6.2)	753(8.9)
	Normal weight	10,719(77.6)	10,646(77.2)	11,274(76.1)	3,914(68.3)	3,902(67.9)	4,325(68.0)	6,805(84.2)	6,744(83.6)	6,949(82.3)
	Overweight	1,569(11.3)	1,617(11.7)	1,622(10.9)	962(16.7)	1,011(17.6)	1,115(17.5)	607(7.5)	606(7.5)	507(6.0)
	Obesity	585(4.2)	593(4.3)	703(4.7)	431(7.5)	406(7.0)	472(7.4)	154(1.9)	187(2.3)	231(2.7)
1000 m/800 m	Excellent	173(1.3)	272(2.0)	158(1.1)	75(1.3)	83(1.4)	50(0.7)	98(1.2)	189(2.3)	108(1.2)
	Good	655(4.7)	1,038(7.5)	449(3.0)	233(4.0)	319(5.5)	173(2.7)	422(5.2)	719(8.9)	276(3.2)
	Pass	9,308(67.4)	9,799(71.1)	8,485(57.3)	3,396(59.2)	3,821(66.5)	3,013(47.3)	5,912(73.1)	5,978(74.3)	5,472(64.8)
	Fail	3,674(26.6)	2,672(19.4)	5,708(38.6)	2,026(35.3)	1,517(26.4)	3,124(49.1)	1,648(20.3)	1,155(14.3)	2,584(30.6)
Total Score	Excellent	13(0.1)	17(0.1)	50(0.3)	9(0.1)	10(0.1)	39(0.6)	4(0.1)	7(0.1)	11(0.1)
	Good	610(4.4)	986(7.1)	764(5.1)	243(4.2)	372(6.4)	320(5.0)	367(4.5)	614(7.6)	444(5.2)
	Pass	10,698(77.4)	11,202(81.2)	12,395(83.7)	4,090(71.3)	4,392(76.5)	4,832(75.9)	6,608(81.7)	6,810(84.6)	7,563(89.6)
	Fail	2,489(18.0)	1,576(11.4)	1,591(10.7)	1,388(24.2)	966(16.8)	1,169(18.3)	1,101(13.6)	610(7.5)	422(5.0)

The endurance running test results revealed that college students’ performance was clustered near the passing threshold (Table 3). The overall passing rate increased from 67.4% in 2018 to 71.1% in 2019, followed by a sharp decline to 57.3% in 2020. A similar trendwas observed in both male and female students. Among males, the passing rate rose from 59.2% to 66.5% in 2019, but dropped markedly to 47.3% in 2020. For females, the rate increased slightly from 73.1% to 74.3% in 2019, but fell to 64.8% in 2020. The failure rate fluctuated over the three years, decreasing from 26.6% in 2018 to 19.4% in 2019, before rising sharply to 38.6% in 2020. Notably, the failure rate was consistently higher among male students than females: 35.3% vs. 20.3% in 2018, and 49.1% vs. 30.6% in 2020, indicating a widening gender gap over time. The proportions of students achieving “good” and “excellent” grades were relatively low, accounting for 5.1% and 1.5% of the total, respectively. Male students were underrepresented in these top two categories compared to females (good: 4.1% vs. 5.8%, excellent: 1.1% vs. 2.1%).

The overall physical fitness test scores (Table 3) revealed that the majority of college students (80.8%) achieved a passing grade. Additionally, 13.0% of students failed, 5.5% were rated as “good,” and 0.17% attained an “excellent” grade. When comparing performance by gender, female students slightly underperformed males in the “excellent”category (0.1% vs. 0.3%). However, females outperformed males in the remaining three categorie: a higher proportion of females students received “good” (5.8% vs. 5.2%), “pass” (85.3% vs. 74.6%), and “fail” grades (8.7% vs. 19.8%).

**Fig 1:**
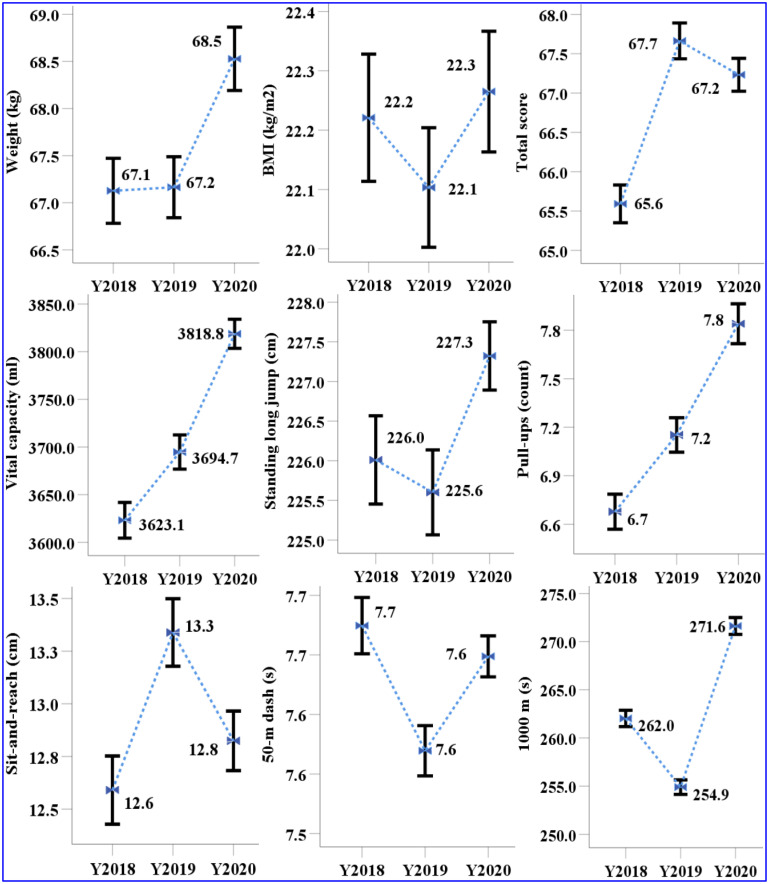
The mean and 95% CI of items of nine physical fitness indicators on male from 2018 to 2020.

**Fig 2:**
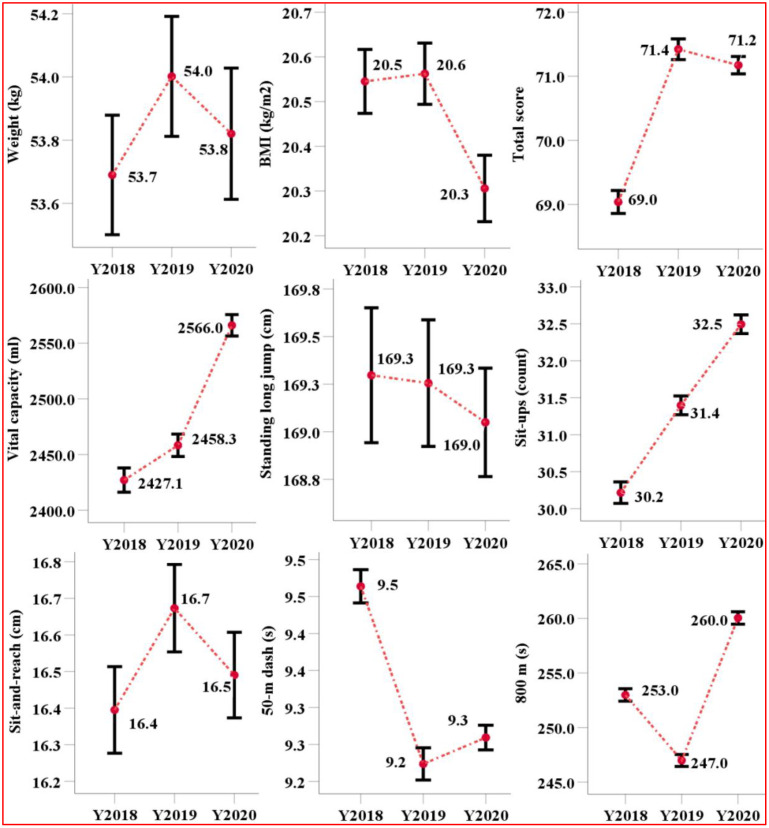
The mean and 95% CI of items of nine physical fitness indicators on female from 2018 to 2020.

Figs 1 and 2 presented the mean values and 95% confidence intervals for nine physical fitness indicators measured from 2018 to 2020, stratified by male and female students. For both genders, the total score, sit-and-reach, 50-m dash, and endurance running exhibited a pattern of initial improvement followed by decline. For males, the total score increased by 2 points and then decreased by 0.4 points; sit-and-reach improved by 0.7 cm before decreasing by 0.5 cm; and the 50-m dash time improved by 0.1 s before increasing again by 0.1 s. Similarly, for females, the total score increased by 2 points and then decreased by 0.3 points; sit-and-reach improved by 0.3 cm before decreasing by 0.2 cm; and the 50-m dash improved by 0.2 s before decreasing slightly by 0.04 s.

In constrast, both vital capacity and upper limb strength (pull-ups for males and sit-ups for females) demonstrated consistent improvements over period. Male students showed an increase in vital capacity of 72.0 ml in 2019 and 24.0 ml in 2020, while pull-up performance improved by 0.5 units in 2019 and 0.6 units in 2020. Female students exhibited an increase in vital capacity of 31.0 ml in 2019 and 108.0 ml in 2020, with sit-ups improved by 1.2 units in 2019 and 1.0 unit in 2020. Regarding the standing long jump, minimal change was oberseved among female students, whereas male students showed a decrease of 0.4 cm followed by an increase of 1.7 cm.

In endurance running, male students improved their running time by 7.1 s in 2019, while female improved by 6.0 s. In 2020, however, performance declined markedly, with running times increasing by 16.7 s and 13.2 s for male and female students, respectively. With the exception of male BMI and female standing long jump scores, all other physical indicators showed statistically significant differences between genders in 2019 and 2020.

## Discussion

The BMI distribution among college students was as follows: normal weight accounted for 77.0%, overweight for 11.3%, underweight for 7.2%, and obesity for 4.4%. The proportion of female students with normal weight (83.4%) was significantly higher than that of males (68.1%). These findings align with those reported by Xia et al. [26] and Sun et al. [27]. However, the proportions of overweight (17.3%) and obesity (7.3%) were considerably higher among male students compared to females (overweight: 7.0%, obesity: 2.3%), which is consistent with the results of Sun et al. [27]. The similarity in these findings may be attributed to the substantial impact of the pandemic on university students overall, with male students being more adversely affected—both on campus and at home—due to their typically higher engagement in outdoor physical activities. Stringent restrictions on group activities during the pandemic significantly reduced opportunities for exercise, which likely contributed to the increased prevalence of overweight and obesity among male students.

From 2018 to 2020, significant differences were observed between male and female participants in height, body mass, upper limb strength (pull-ups for males, sit-ups for females), endurance running (1000 m for males, 800 m for females), and overall physical fitness scores. Notably, vital capacity and upper limb strength showed a continuous upward trend during this period for both genders. Specifically, male students exhibited increases in vital capacity by 2.0% and 3.4%, and in upper limb strength by 4.8% and 8.3%, over the two intervals, respectively. Similarly, female students showed improvements in vital capacity by 1.3% and 4.4%, and in upper limb strength by 3.9% and 3.5%. These findings are consistent with those reported by Fearnbach et al. [28]. Xia et al. also observed similar improvements and suggested that these gains may be attributed to the straightforward, convenient, and practical implementation of relevant interventions [26]. Similarly, Sun et al. proposed that the rationale for delivering targeted exercises through online physical education served as an effective guidance under quarantine conditions [27].

Notably, in the endurance running event, both genders exhibited a similar trend from 2018–2020: an initial improvement followed by a subsequent decline. Specifically, performance improved in 2019, with times decreasing by 7.1 s for males and 6.0 s for females. However, in 2020, performance declined markedly, with times increasing by 16.7 s for males and 13.2 s for females. This pattern suggests that the short-term impact of the pandemic had not yet fully materialized during its initial outbreak, when containment measures were still in the early stages of implementation and the situation was highly unpredictable. By the fall of 2020, however, the pandemic had persisted for nearly two years, and its profound effects on students’ lives and learning had become fully apparent—particularly the irreversible impact on endurance running performance. The decline in endurance running is consistent with observations reported by Xia et al. and Sun et al. [26,27]. These findings highlight the delayed yet substantial long-term effects of the pandemic, which significantly altered both lifestyle and learning patterns among college students.

Feng et al. attributed the decline in endurance running and pull-up performance among male students at Tsinghua University to reduced exercise volume and intensity resulting from online physical education during the pandemic [29], a view consistent with the changes in learning modalities observed in this study among university students. In contrast, our findings suggest that the COVID-19 pandemic had a more pronounced effect on endurance running compared to upper limb strength [30–32]. With the exception of minor variations in male BMI and female standing long jump performance, all other physical fitness indicators showed significant gender differences in both 2019 and 2020—results that align with those reported by Xia et al. [26], who attributed the decline primarily to insufficient running practice and cancellation of athletic events due to venue inaccessibility during the pandemic. These results further underscore the substantial impact of the pandemic on college students’ physical fitness. On one hand, pandemic restrictions limited opportunities for physical activity in both time and space, preventing students from engaging in long-distance running practice on tracks, which led to a sharp decline in cardiopulmonary function—reflecting a drastic change in lifestyle [5,33–36]. On the other hand, psychological stress and anxiety associated with the pandemic may have compounded its short-term effects on students’ physical health [37–41].

In the endurance running event, the proportion of males students in both the “good” and “excellent” categories was lower than that of females, a trend aligns with the findings reported by Xia et al. [26]. The widespread epidemic led to the implementation of various lockdown and control measures by governments and education institutions [42–45]. Consequently, students were largely confined to their dormitories, and outdoor physical education classes were completely canceled due to significant alterations in how physical education was delivered [46–48]. This lack of opportunity for effective physical exercise compromised students’ ability to maintain normal cardiopulmonary function [49–51], resulting in diminished aerobic endurance and substantial decline in long-distance endurance capabilities [52,53].

From 2018 to 2020, the average overall physical fitness score of female students at Chizhou University (70.5) was higher than that of males’ (66.8), a trend also observed at Shenyang medical college [54]. This study revealed that most college students at Chizhou University scored near the pass mark of 60.0 (80.8%), while 13.0% failed to meet thestandard, 5.5% were rated as “good” status, and only 0.2% achieved “excellent” status. With the exception of the “excellent” category, where male slightly outperformed females, female students demonstrated better performance across the remaining three categories. In recent years, the physical fitness of college students has generally been unsatisfactory. Research suggests that physical fitness has been steadily declining annually [55–57], associated with factors such as increased sedentary behavior [58–60], greater reliance on take-out food [13], and prolonged screen time [21]. These behavioral shifts reflect significant changes in lifestyle and study habits among students resulting from the pandemic.

The physical fitness scores for both males and females, including performance in sit-and-reach, 50-m dash, and endurance running events, exhibited a trend of initial improvement followed by decline. This trend differs somewhat from the continuous upward trajectory reported by Xia et al. and Sun et al. [26,27]. The COVID-19 epidemic has compelled significant changes in daily lifestyles and methods of sports training [61,62]. Although college students continued to engage indoors exercise according to teachers’ instructions and video guidance, yet the pandemic has still had a notable impact on their physical flexibility and short-distance speed events. Meanwhile, there has been a growing recognition of health-related concepts such as “exercise promotes health” and “exercise is medicine” [63–66].

In this study, vital capacity and upper limb strength (measured by pull-ups and sit-ups) showed consistent year-over-year improvements, a finding aligns with other studies [26,27]. In terms of sit-ups, females showed an increase of 1.2 repetitions in 2019 and 1.0 in 2020, which consistent with Xia et al. (1.0) and Sun et al.(2.0) in 2020 [26,27]. These improvements may be attributed to the adapted physical education implemented during the COVID-19 epidemic. Instructors emphasized organizing indoor sports activities and encouraged students to independently achieve exercise goals [27,53,67]. Amid the rapid spread of the virus, both teachers and students became acutely aware of the importance of physical health in combating COVID-19. Although outdoor activities were restricted, this heightened awareness and increased engagement in indoor exercises led to a noticeable improvement in upper limb strength, indicating a significant increase in college students’ upper body power during the epidemic [15]. In contrast, standing long jump performance showed only marginal improvement among female students, though male students exhibited increased performance in 2020. This finding partially diverges from those of Xia et al. and Sun et al., who reported improvements in both genders [26,27]. The moderate gains in standing long jump may be due to greater focus exercises targeting cardiopulmonary function and upper limb strength, with comparativley less emphasis on lower limb training. These observations underscore the importance of developing well-rounded and scientifically structured fitness programs to promote balanced physical health development [68,69].

This study focused on the impact of the COVID-19 pandemic on the physical fitness of university students before and after its outbreak. However, several limitations should be acknowledged due to sample size and research capacity. First, the sample was drawn from a single university in southern Anhui Province. Although over 80.0% of the students originated from within the province, the findings may not be representative of the entire Anhui student population, nor can they be generalized to the broader Chinese university student body or other types of institutions (e.g., sports universities or top-tier national universities). Second, although the study emphasized changes in lifestyle and learning patterns, other influencing factors—such as consumption of takeout food, screen time, and psychological status—were not sufficiently measured or controlled. These pandemic-related factors could only be treated collectively as a composite influence. Third, the year 2020 represents only the initial phase of the pandemic. The delayed long-term effects warrant further in-depth investigation to more precisely determine the specific impact of the pandemic.

## Conclusion

The COVID-19 pandemic has had a significant negative impact on the physical fitness of university students, leading to increased rates of overweight and obesity, as well as a decline in endurance running performance. It is worth noting, however, that vital capacity and upper limb strength showed a continuous upward trend during this period. The underlying mechanisms for these divergent responses warrant further investigation. Future efforts should focus on the sustained effects of pandemic-induced changes in lifestyle and learning behaviors—such as increased consumption of takeout foods, excessive screen time, and psychological health issues—which may have long-term and delayed consequences on physical fitness. Additionally, there is an urgent need to develop and implement alternative exercise modalities during major public health emergencies to help maintain and promote continuous physical health.

## Supporting information

S1 Table Summary statistics for other measuredphysical fitness metrics. (XLS)

S1 Data (XLS)
